# Supplementary material for: Immunohistochemical Evaluation of Candidate Biomarkers for Fluorescence-Guided Surgery of Myxofibrosarcoma Using an Objective Scoring Method
Source: Biomedicines. 2023 Mar 22;11(3):982. doi: 10.3390/biomedicines11030982 (PMC10046284; doi:10.3390/biomedicines11030982)
Supplement: Supplementary file 1 [file biomedicines-11-00982-s001.zip › biomedicines-2218601-supplementary.pdf]

Supplementary Table S1. Antibodies used for immunohistochemical evaluation.

Antigen	Source	Clone	Manufacturer	Cat. Nr.	Positive control	Negative control	Dilution	Retrieval buffer	Retrieval method	Incubation temperature
TEM-1	Rabbit monoclonal	EPR17081	Abcam	204914	Breast and endometrium	Spleen	1:2000	pH high (EDTA)	Overnight	4°C
VEGFR-1	Rabbit monoclonal	Y103	Abcam	32152	Placenta	Liver	1:500	pH low (citrate)	Overnight	4°C
VEGFR-2	Rabbit monoclonal	55B11	Cell signaling technology	2479	Placenta and kidney	Skin	1:300	pH high (EDTA)	Overnight	4°C
VEGF-A	Mouse monoclonal	G153-694	BD Pharmingen	555036	Placenta and tonsil	Colon	1:400	pH low (citrate)	1 hour	RT
EGFR	Rabbit monoclonal	D38B1XP	Cell signaling technology	4267	Placenta	Tonsil	1:300	pH low (citrate)	1 hour	RT
IGF-1R	Rabbit monoclonal	D406W	Cell signaling technology	14534	Placenta	Skin	1:200	pH low (citrate)	Overnight	RT
PDGFR-α	Rabbit monoclonal	D13C6	Cell signaling technology	5241	Placenta and Skin	Spleen	1:100	pH high (EDTA)	Overnight	4°C
CD40	Rabbit monoclonal	D8W3N	Cell signaling technology	40868	Tonsil	Skin	1:100	pH high (EDTA)	Overnight	4°C

Abbreviations: TEM-1 = Tumor endothelial marker-1; VEGFR-1 = Vascular endothelial growth factor receptor-1; VEGFR-2 = Vascular endothelial growth factor receptor-2; VEGF-A = Vascular endothelial growth factor-A; EGFR = Epidermal growth factor receptor; IGF-1R = Insulin-like growth factor-1 receptor; PDGFR-α = Platelet derived growth factor receptor α; CD 40 = Cluster of differentiation 40; and RT = Room Temperature.

Supplementary Protocol S1. Objective immunohistochemistry scoring method.

Step 1. Select the regions of interest.

Open the stained slide in ImageJ and select the regions of interest based on annotations from the pathologist. In ImageJ use "Analyze -> Tools -> ROI manager…" to select tumor tissue and adjacent healthy tissue (Figure S1).

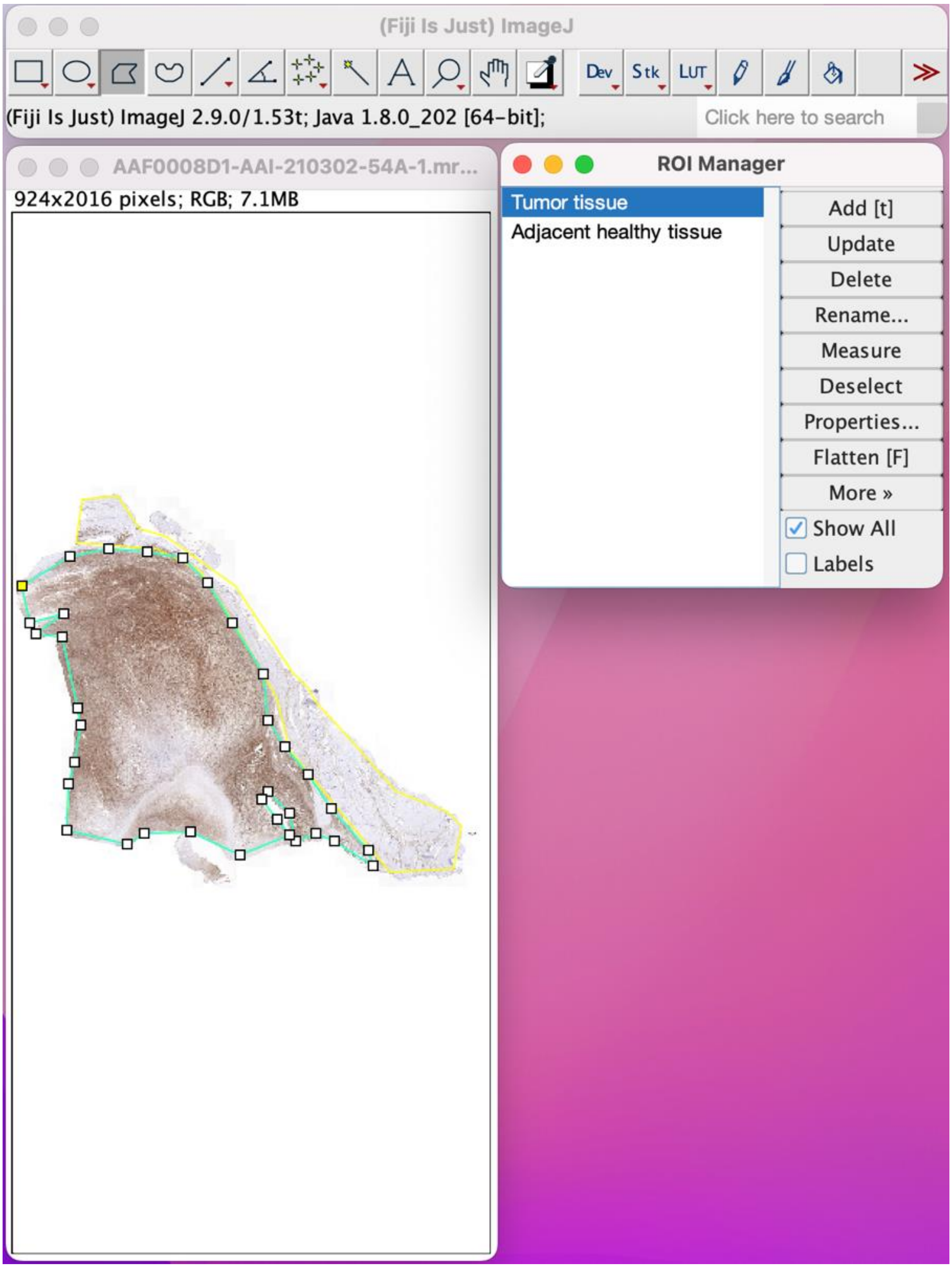

Figure S1. The regions of interest "Tumor tissue" and "Adjacent healthy tissue" have been selected.

Step 2. Filter out background colors using the "color deconvolution" option in ImageJ.

Select “Image -> Color -> Color deconvolution and select H&E DAB” in ImageJ to acquire the image that only shows the relevant brown DAB staining (Figure S2).

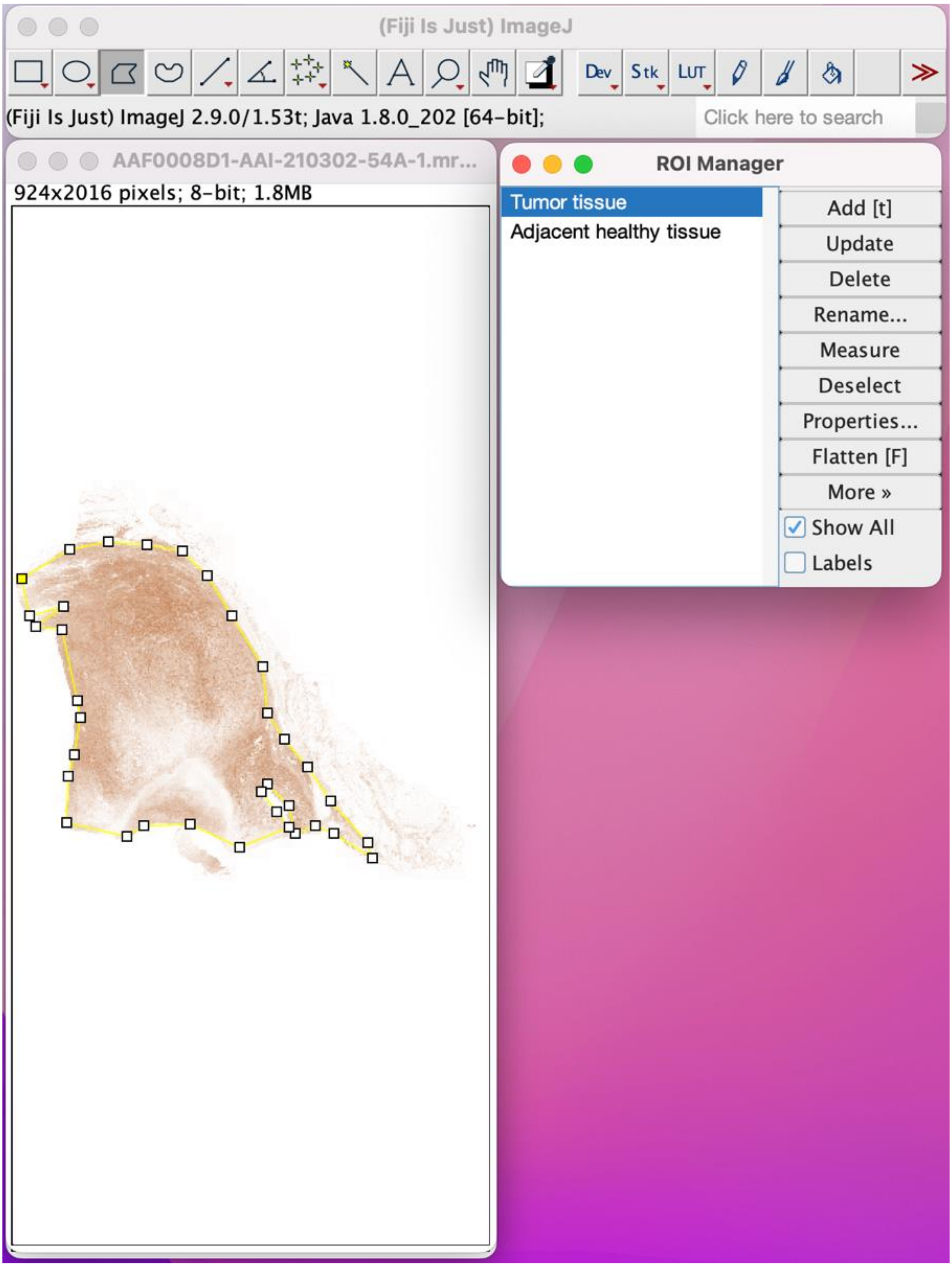

Figure S2. Only the relevant DAB staining is selected.

Step 3. Change color to greyscale

Select “Edit -> Options -> Conversions…” and tick the “Weighted RGB conversions” box and click “OK”. Afterwards, select “Image -> Type -> 8-bit” to convert the brown DAB staining into grayscale.

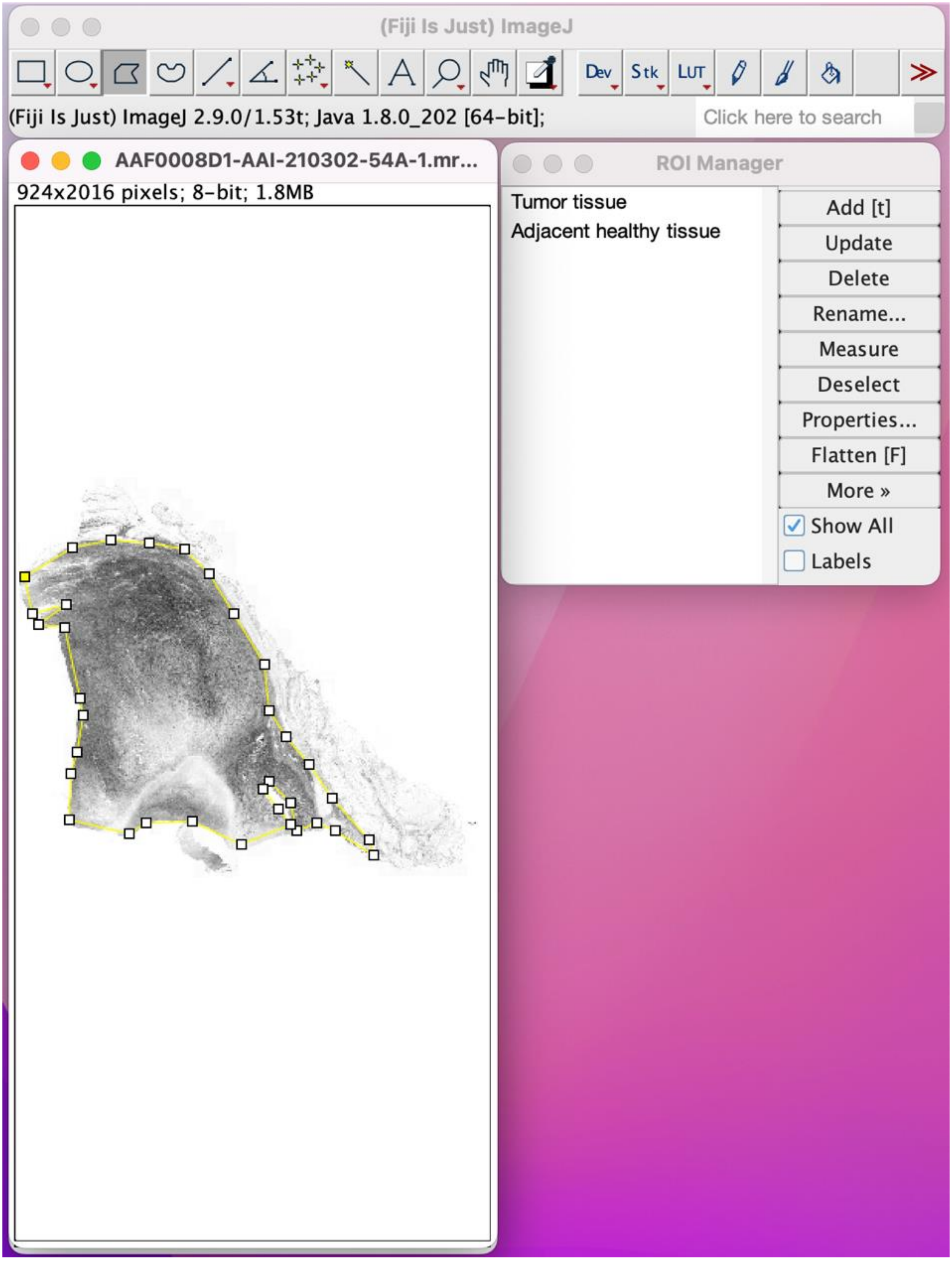

Figure S3. The image is converted into grayscale.

Step 4. Invert black and white

Select "Edit-> Invert" to invert the image. Now the pixel values correlate with staining intensity (Value black = 0 and value white = 255; Figure S4).

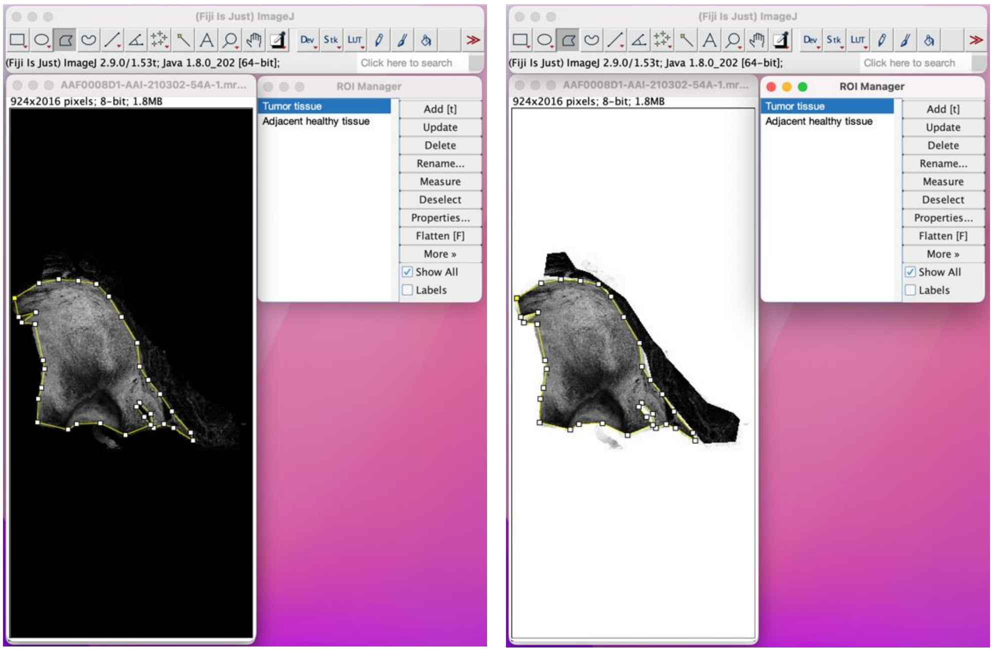

Figure S4. The whole image is inverted (left) or the selected regions are inverted (right); both options give the same result.

Step 5. Measure the mean pixel (staining) intensity value and the standard deviation
Select "Analyze -> Set Measurements…" and select "Mean gray value and Standard deviation". In ROI manager select measure (Figure S5).

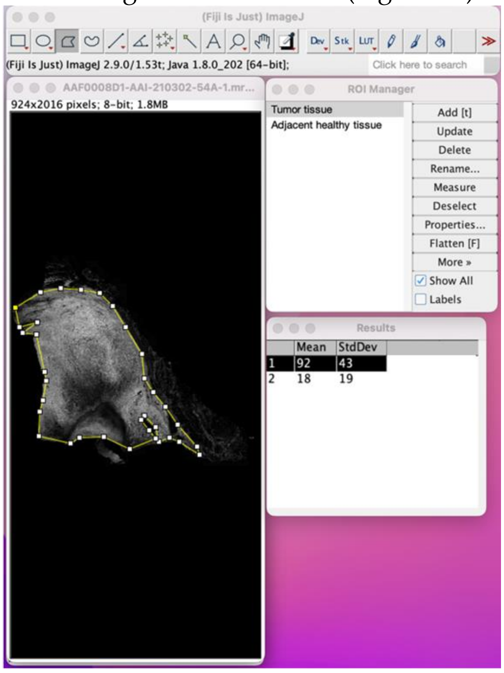

Figure S5. Mean intensity and standard deviation intensity values have been measured in "Tumor tissue (1)", and "Adjacent healthy tissue (2)".

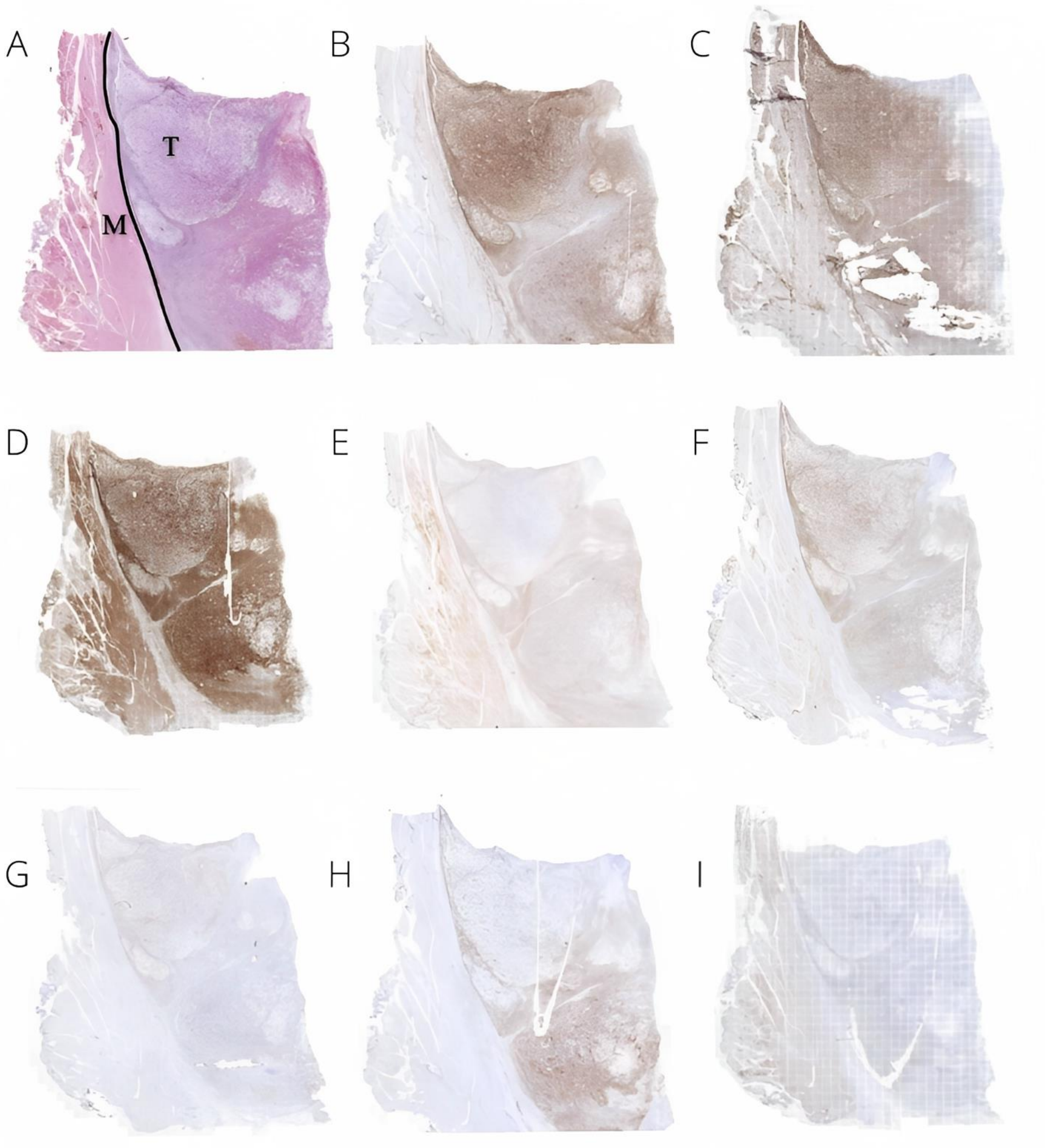

Supplementary Figure S6. MFS tumor located in the lower arm of a 64-year-old male who received preoperative radiotherapy. H&E staining of MFS tumor tissue (T), the tumor border (black line) and adjacent muscle tissue (M) (A). Corresponding TEM-1 staining displays TEM-1 expression in MFS tumor tissue, while TEM-1 expression in adjacent muscle tissue was low (B). PDGFR-α and VEGF-A staining was strong in MFS tumor tissue, but adjacent muscle tissue was also stained positive (C, D). Expression of VEGFR-2, VEGFR-1, EGFR, CD40 and GF-1R was less evident in MFS tumor tissue (E up untill I).

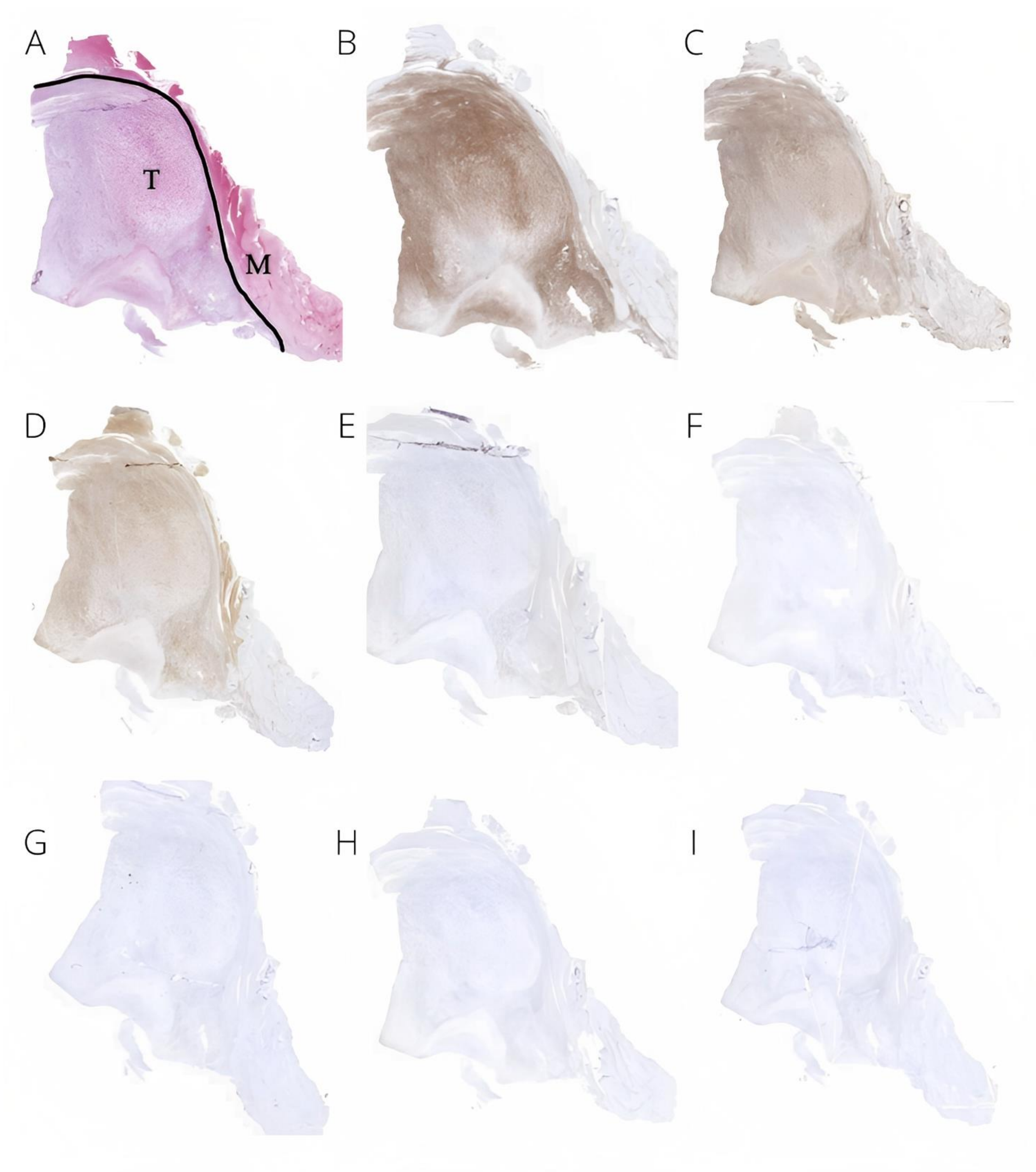

Supplementary Figure S7. Grade 2 MFS tumor located in the upper arm of a 63-year-old female. H&E staining of MFS tumor tissue (T), the tumor border (black line) and adjacent muscle tissue (M) (A). Corresponding TEM-1 staining shows strong TEM-1 expression in MFS tumor tissue, while TEM-1 expression in adjacent muscle tissue was low (B). PDGFR-α and VEGF-A staining was strong in MFS tumor tissue, but adjacent muscle tissue also stained positive (C, D). Although positive controls stained positive, there was virtually no expression of VEGFR-2, VEGFR-1, EGFR, CD40 and IGF-1R in MFS tumor tissue (E up untill I).

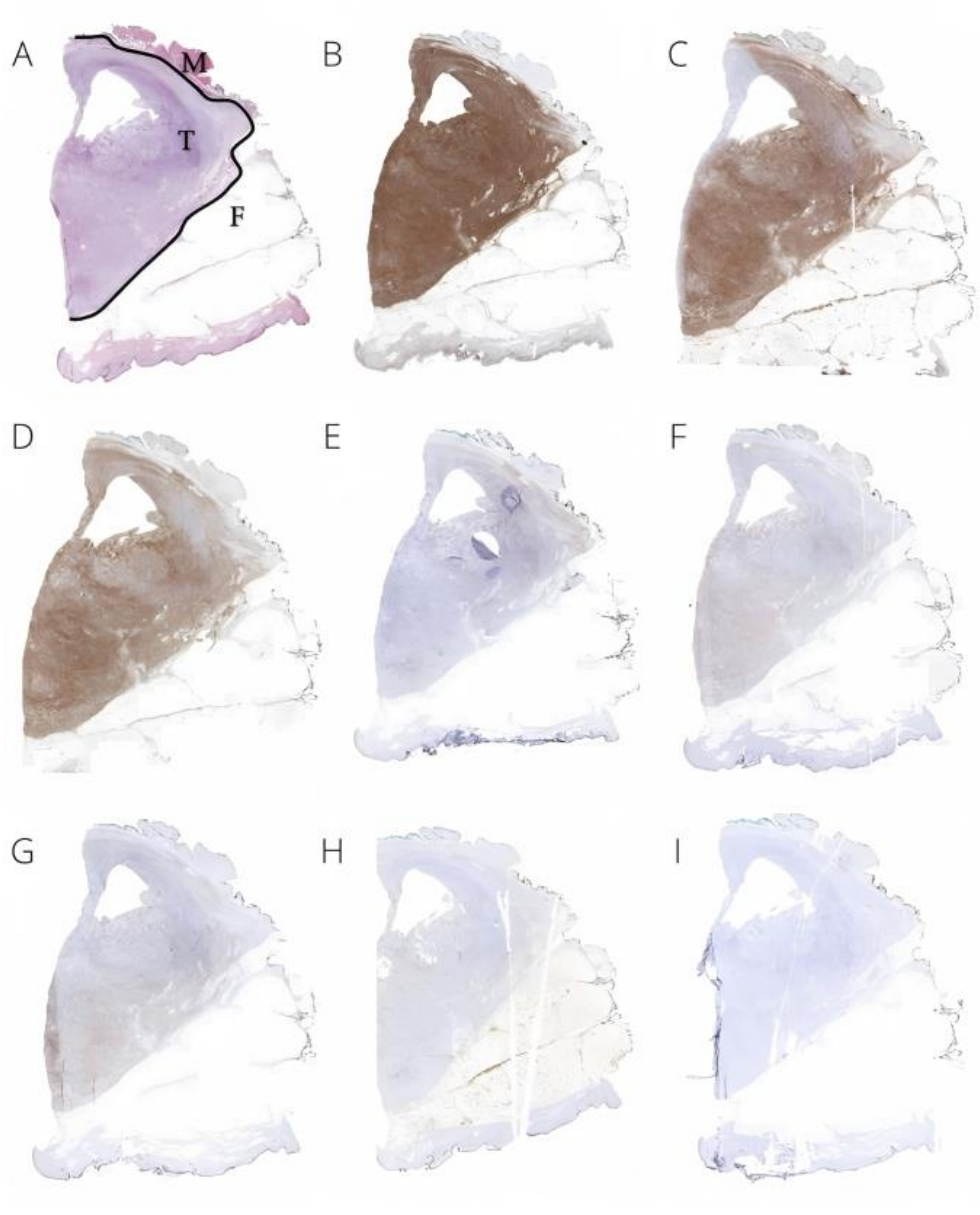

Supplementary Figure S8. Grade 3 MFS tumor located in the upper arm of a 64-year-old female. H&E staining of MFS tumor tissue (T), the tumor border (black line) and adjacent muscle- and fat tissue (M and F) (A). Corresponding TEM-1 staining displays TEM-1 expression in MFS tumor tissue, while TEM-1 expression in adjacent muscle- and fat tissue was low (B). PDGFR-α and VEGF-A staining was also strong in MFS tumor tissue, with little staining in adjacent muscle- and fat tissue (C, D). There was virtually no expression of VEGFR-2, VEGFR-1, EGFR, CD40 and IGF-1R (E up untill I).
